# Microplastics, nanoplastics, and thrombogenesis

**DOI:** 10.1016/j.rpth.2026.106931

**Published:** 2026-08-28

**Authors:** Rhesha Balwan, Yu Yue Hew, Gerard Gurumurthy, Xiao Wei Ang, Ryan Ruiyang Ling, Chuanhui Xu, Ying Jie Chee, Rinkoo Dalan, Prahlad Ho, Hui Yin Lim, Barbara D. Lam, Jecko Thachil, Emmanuel J. Favaloro, Bingwen Eugene Fan

**Affiliations:** 1 Department of Haematology, Tan Tock Seng Hospital, Singapore; 2 The Queen Elizabeth Kings Lynn National Health Service Foundation Trust, Kings Lynn, United Kingdom; 3 Addenbrooke’s Hospital, Cambridge University Hospitals National Health Service Foundation Trust, Cambridge, United Kingdom; 4 Yong Loo Lin School of Medicine, National University of Singapore, Singapore; 5 Australia and New Zealand Intensive Care Research Centre, School of Public Health and Preventive Medicine, Monash University, Melbourne, Victoria, Australia; 6 Lee Kong Chian School of Medicine, Nanyang Technological University, Singapore; 7 Department of Rheumatology, Allergy and Immunology, Tan Tock Seng Hospital, Singapore; 8 KLARIS Precision Health Research Clinic, Tan Tock Seng Hospital, Singapore; 9 Department of Endocrinology, Tan Tock Seng Hospital, Singapore; 10 Northern Clinical Diagnostics and Thrombovascular Research Centre, Northern Health, Epping, Victoria, Australia; 11 Department of Medicine, Northern Health, University of Melbourne, Epping, Victoria, Australia; 12 School of Health and Biomedical Sciences, Royal Melbourne Institute of Technology University, Bundoora, Victoria, Australia; 13 Division of Hematology & Oncology, Fred Hutch Cancer Center, University of Washington, Seattle, Washington, USA; 14 Manchester Academic Health Science Centre, The University of Manchester, Manchester, United Kingdom; 15 Haematology Department, Institute of Clinical Pathology and Medical Research, New South Wales Health Pathology, Westmead Hospital, Westmead, New South Wales, Australia; 16 Sydney Centres for Thrombosis and Haemostasis, Research and Education Network, Institute of Clinical Pathology and Medical Research, Westmead Hospital, Westmead, New South Wales, Australia; 17 School of Dentistry and Medical Sciences, Faculty of Science and Health, Charles Sturt University, Wagga Wagga, New South Wales, Australia; 18 School of Medical Sciences, Faculty of Medicine and Health, University of Sydney, Westmead Hospital, Westmead, New South Wales, Australia

**Keywords:** endothelial dysfunction, hypercoagulability, microplastics, nanoplastics, thrombosis

## Abstract

Micro- and nanoplastics (MNPs) are produced through mechanical, chemical, and ultraviolet-mediated degradation of plastics and may enter the human bloodstream via ingestion, inhalation, dermal exposure, and iatrogenic sources. Their detection in human blood, atheromas, and thrombi has raised concerns that they may contribute to thrombogenesis. We conducted a structured narrative review of English-language studies indexed in PubMed, Embase, and Scopus over the preceding 15 years, up to March 31, 2026. The search identified 981 database records and 39 additional manually identified articles; after removing duplicates and screening, 72 articles were included. Evidence was synthesized according to Virchow’s triad, focusing on endothelial injury, altered blood flow, hypercoagulability, cellular uptake, polymer type, surface charge, weathering, and protein corona formation. MNPs may contribute to all 3 components of Virchow’s triad. Proposed mechanisms include oxidative endothelial injury, glycocalyx disruption, monocyte adhesion, foam-cell formation, plaque instability, immune cell-mediated microvascular obstruction, platelet activation, NETosis, von Willebrand factor interactions, factor XII activation, thrombin generation, and fibrin-rich clot stabilization. These effects appear to vary with particle size, polymer composition, surface charge, and environmental weathering. However, mechanistic evidence largely comes from polystyrene nanoparticle models, whereas human thrombus and atheroma studies more commonly detect polyethylene and polyvinyl chloride. MNPs are biologically plausible contributors to thrombogenesis, but their clinical relevance remains uncertain. Prospective human studies, standardized contamination-controlled detection methods, and dose-response experiments using clinically relevant particles and concentrations are required.

## Essentials

• Micro- and nanoplastics are increasingly detected in human blood and thrombi.
• This review evaluates their possible role in thrombosis using Virchow’s triad.
• Microplastics induce inflammation, platelet activation, and endothelial injury, which may promote thrombosis.
• Clinical relevance requires validated assays, dose-response data, and longitudinal studies.

## 1 Introduction

The global annual plastic production increased from 2 megatons in 1950 to 475 megatons in 2022 and is projected to rise to 749 to 976 megatons in 2050 [1]. Through mechanical wear, chemical breakdown, and UV radiation, plastics fragment into microplastics (MPs; 0.1 μm to 5 mm in diameter) and nanoplastics (NPs; <0.1 μm in diameter) [2]. MPs and NPs (MNPs) can infiltrate human blood and may contribute to thrombogenicity. They enter the body through inhalation of airborne fibers shed from synthetic clothing, ingestion of contaminated food or water, or dermal penetration [3,4]. Atmospheric MNPs act as vectors by adsorbing airborne chemicals, such as pesticides, that enter our bodies through inhalation [5]. Iatrogenic MP exposure introduces polymer-derived particles into the systemic circulation. Sterilization, exposure to high temperatures or UV light, improper storage, and mechanical friction may promote surface degradation and MP particle formation. These particles may enter the bloodstream intravenously or come into direct contact with body cavities through devices such as catheters [6]. According to Cox et al. [7], the estimated annual human intake of MPs is 74,000 to 121,000 particles per year.

Since MNPs directly interact with endothelial and immune cells to induce platelet aggregation, they may contribute to thrombus formation [8]. Several mechanisms, including inhibition of immune cell function, mechanical obstruction, formation of neutrophil extracellular traps (NETs), platelet activation, chronic inflammation, and endothelial adhesion, have been postulated [9,10]. MNPs have been detected in human thrombi retrieved from patients with ischemic stroke, myocardial infarction, and deep vein thrombosis [10]. Given this, it is biologically plausible that MNPs could influence thrombotic pathways. Therefore, this literature review summarizes the proposed mechanisms by which MNPs may contribute to thrombogenicity.

## 2 Methods

A structured narrative literature review was conducted to identify studies examining the relationship between MPs, NPs, polymer particles, and thrombogenic pathways. PubMed, Embase, and Scopus were searched for English-language articles published in the preceding 15 years, up to March 31, 2026. The primary search combined controlled vocabulary terms, where available, and free-text terms related to particle exposure (“microplastic,” “nanoplastic,” “plastic particle,” “polymer particle,” “secondary microplastic,” “primary microplastic,” “micro polymer,” and “nano polymer”) with thrombosis-related terms (“embol∗,” “thrombosis,” “thrombus,” “thrombi,” “thrombotic,” and “thromboembol∗”). The full database search strategy is provided in the Supplementary Methods. Additional targeted searches were performed to identify mechanistic literature relevant to NETosis, platelet activation, chronic inflammation, endothelial adhesion, surface-modified MNPs, endothelial damage, blood flow alteration, and hypercoagulability. The reference lists of included articles and relevant reviews were also manually screened to identify additional studies.

Studies were eligible for inclusion if they examined the presence, biological effects, or mechanistic relationships of MPs, NPs, or polymer particles with thrombosis, coagulation, platelet activation, endothelial dysfunction, inflammation, altered blood flow, vascular obstruction, or related thromboinflammatory pathways. *In vitro*, *in vivo*, and human observational studies were considered. Articles were excluded if they were not available in English, did not address vascular or thrombotic mechanisms, or were not directly relevant to the conceptual framework of this review. Titles, abstracts, and full texts were screened by 2 independent reviewers (R.B. and Y.Y.H.), with discrepancies resolved through discussion and, when necessary, consultation with a third reviewer (B.E.F.).

Database searches identified 981 records, and 39 additional articles were identified through manual searching. After duplicate removal and screening, 72 articles were included in the final narrative synthesis (Figure 1). These were grouped by primary contribution: background and exposure literature; cellular uptake of MNPs; mechanistic evidence mapped to Virchow’s triad; evidence of MNPs in human blood or thrombi; surface modification as a determinant of biological effect; and others (Supplementary Table). Given the central role of Virchow’s triad in thrombogenesis, we used it as the organizing framework for this review and examined how MNPs may contribute to endothelial injury, altered blood flow, and hypercoagulability. Since this study was designed as a structured narrative mechanistic review rather than a formal systematic review or meta-analysis, no quantitative synthesis was performed.

**Figure 1:**
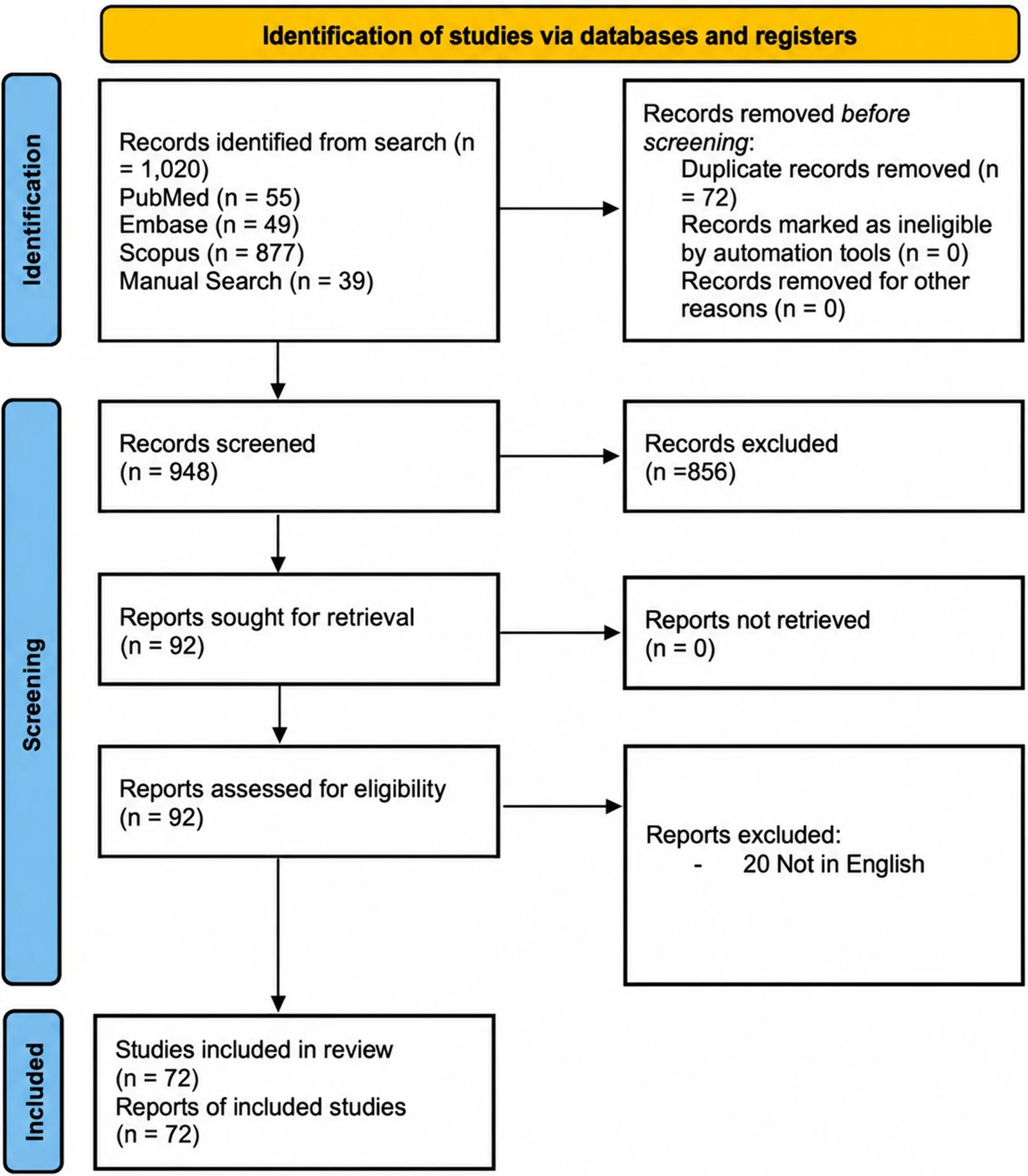
Preferred Reporting Items for Systematic reviews and Meta-Analyses (PRISMA) diagram of the literature review.

## 3 Results

### 3.1 Cellular uptake of MNPs

The ability of MNP particles to translocate into systemic circulation is inversely related to particle size [11]. NPs (∼40 nm) are phagocytosed by macrophages, whereas larger MPs (∼6.8 μm) can cause mechanical blockage of capillaries [12]. When macrophages engulf larger NP aggregates, they undergo restructuring of their internal actin cytoskeleton [13] and lose flexibility, potentially causing mechanical obstruction of the capillary wall. Cellular internalization of MNPs begins with cell membrane adhesion, followed by passive penetration or active endocytosis [14]. The specific endocytic mechanism varies by particle size: smaller NPs (∼60-120 nm) are often internalized via caveolae and clathrin-mediated endocytosis, while MPs (≥500 nm) require actin-dependent uptake, like phagocytosis [15].

Only NPs and small MPs (∼5-10 μm) generally remain in circulation in the blood, whereas larger particles tend to be sequestered in the capillaries [16]. Vlacil et al. [17] reported that polystyrene (PS) NPs activate the endothelium through monocyte adhesion, leading to the formation of a subsequent atherosclerotic plaque that eventually ruptures and releases prothrombotic factors. Additionally, the uptake of MNPs by neutrophils and monocytes induces apoptosis [18]. Interestingly, carboxylated PS (cPS) NPs have superior cellular uptake, reactive oxygen species (ROS) generation, and apoptosis induction compared with nonfunctionalized PS (nPS) NPs, aminated PS (aPS) NPs, and micronized PS NPs [19]. NPs can also cross the blood-brain barrier. Huang et al. [20] found that obstruction by MP-laden immune cells caused other MP-laden cells to coalesce, increasing the obstruction area. Platelets may adhere and aggregate at the obstruction site, leading to thrombus formation.

### 3.2 Virchow’s triad

Thrombogenesis may be attributed to Virchow’s triad, which comprises endothelial damage, altered blood flow, and hypercoagulability.

#### 3.2.1 Endothelial damage

MNP exposure increases vascular endothelial adhesiveness. According to a study by Wei et al. [21], transwell assays suggest that endothelial leakiness may be triggered by negatively charged PS NPs. NPs directly bind to vascular endothelial cadherin, suggesting that physical disruption of endothelial adherens junctions underlies the induced endothelial leakiness. These intercellular gaps lead to immune cell infiltration into the vessel wall, which becomes a site of inflammation. Overexpression of vascular cell adhesion molecule-1 and intercellular adhesion molecule-1 ensues, to which platelets then adhere. Eventually, a platelet plug forms in the vessel wall, and coagulation is activated [22].

MNPs may promote oxidative stress by generating ROS [23,24]. Photo-oxidation or UV exposure can further alter particle surfaces and increase extracellular free-radical generation after cellular uptake [23]. Oxidative stress may damage the endothelial glycocalyx (EG) and disrupt anticoagulant surface molecules, such as heparan sulfate and thrombomodulin, thereby reducing local thrombin regulation and favoring fibrin formation. Endothelial injury also loosens tight junctions, allowing low-density lipoprotein (LDL) entry into the intima, LDL oxidation, monocyte recruitment, foam cell formation, and atherosclerotic plaque development [25]. Oxidative stress and inflammatory remodeling may weaken the fibrous cap, expose tissue factor, activate the extrinsic coagulation pathway, and promote thrombus formation (Figure 2).

**Figure 2:**
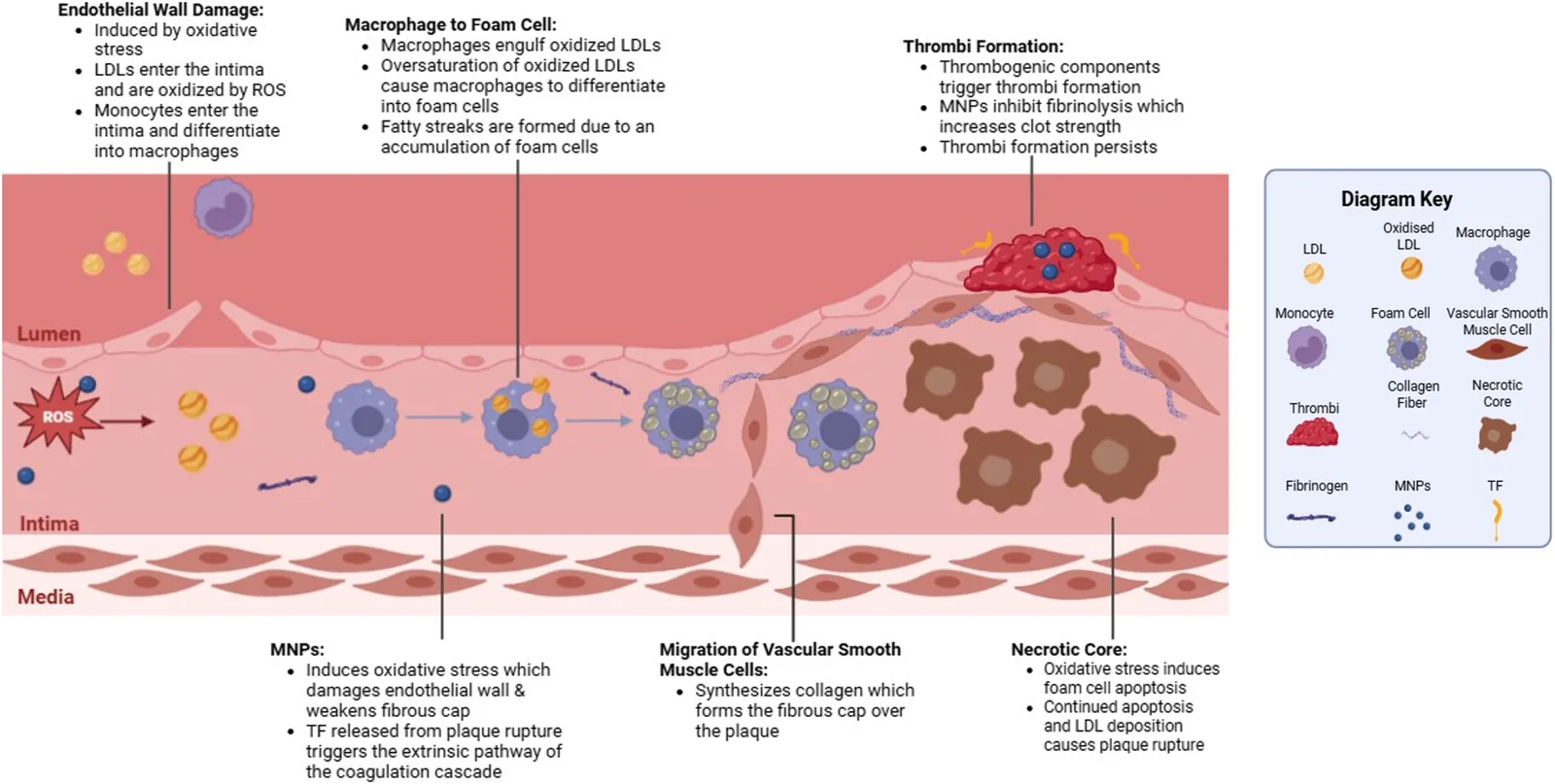
Micro- and nanoplastic (MNP)-associated endothelial injury, atherogenesis, and plaque rupture-driven thrombosis. MNPs may promote oxidative endothelial injury and facilitate low-density lipoprotein (LDL) entry into the intima, LDL oxidation, monocyte recruitment, macrophage foam cell formation, and atherosclerotic plaque development. Oxidative stress and inflammatory remodeling may weaken the fibrous cap, exposing thrombogenic plaque components and tissue factor (TF), thereby activating coagulation and promoting thrombus formation. ROS, reactive oxygen species.

Marfella et al. [26] reported that polyethylene (PE) particles were detected in carotid artery plaques in 150 of 304 participants and polyvinyl chloride (PVC) particles in 31 participants. This suggests that MNPs may contribute to atherosclerosis by initially depositing in the subendothelial space. Emerging evidence suggests that MNPs may promote atherosclerosis through a distinct inflammatory mechanism. MNPs are internalized and accumulate in macrophage lysosomes [27], triggering lysosomal membrane damage and increased proinflammatory cytokines [28]. MNPs also increase mitochondrial ROS levels, activate necrosomes, and trigger cell death. The NOD-like receptor family pyrin domain-3 (NLRP3) inflammasome is critical in the innate immune system for secreting proinflammatory cytokines. When activated during apoptosis, NLRP3 engages with the apoptosis-associated speck-like protein, resulting in the proteolytic maturation of caspase-1. Mature caspase-1 converts prointerleukin (IL)-1β and pro–IL-18 into their active forms, IL-1β and IL-18, respectively, thereby inducing pyroptosis [29]. Due to inflammation in macrophages, cells burst and accumulate to form a necrotic core, creating an atherosclerotic plaque. Cytokines released from burst macrophages thin the fibrous cap of the necrotic core, trigger plaque rupture, and cause thrombogenesis [26].

#### 3.2.2 Blood flow alteration

Once in the bloodstream, MNPs are phagocytosed by neutrophils and macrophages [30]. One study demonstrated that smaller PS MPs (0.5 μm) had higher internalization rates than larger PS MPs (5 μm) [31]. When macrophages attempt to engulf larger NP conglomerates, roundabout guidance receptor 1-mediated actin cytoskeleton rearrangement is triggered, causing NP-laden macrophages to lose their cellular deformability [13]. As a result, these macrophages become rigid and can mechanically obstruct the capillary wall, slowing blood flow and causing blood to accumulate at the site of obstruction.

Simultaneously, MNPs disrupt blood flow dynamics at the vessel wall by degrading EG (located on the endothelial luminal layer) [32]. An EG unit consists of glycosaminoglycan chains and associated glycoproteins (GPs) [33]. ROS, which impair glycosaminoglycan chains, may alter surrounding blood flow. Jiang et al. [34] demonstrated that glycocalyx molecules alter velocity distribution and increase blood flow shear stress in the near-wall region. In another experiment, a single EG chain was stimulated to shed from the endothelial surface. As the chain tumbled, it struck adjacent water molecules with measurable force (0.1-1 pN per bead). By measuring the orientation of flow vectors (θ), Jiang et al. [35] found that the shed chain significantly increased flow disorder. With an intact EG, flow vectors are predominantly unidirectional. However, shedding introduced random vertical and horizontal velocity components, disturbing the flow field.

#### 3.2.3 Hypercoagulability

When neutrophils ingest MNPs, they may undergo a stress response that causes them to extrude their own DNA, forming NETs [36]. A NET network structure could serve as a scaffold for depositing platelets, erythrocytes, fibrinogen, von Willebrand factor (VWF), and platelet adhesion factors, which contribute to thrombus formation. PS NPs have been shown to activate the Toll-like receptor (TLR) 4/nuclear factor (NF)-κB pathway [37]. Rivadeneyra et al. [38] demonstrated that a TLR4 agonist potentiated thrombin-induced aggregation, fibrinogen binding, and adenosine triphosphate secretion by activating NF-κB, potentially amplifying hypercoagulability.

Christodoulides et al. [39] concluded that negatively charged cPS particles could be a primary driver of hypercoagulability. Moreover, Lunov et al. [40] demonstrated that aPS NPs trigger NLRP3 inflammasome activation and IL-1β release. TLR2/4 receptors also activate proinflammatory signaling, mainly via the NF-κB and mitogen-activated protein kinase downstream signaling pathways [41]. TLR4 promotes NLRP3 inflammasome activation via NF-κB–dependent priming, which upregulates proinflammatory cytokines IL-6, IL-1β, and tumor necrosis factor-α, and promotes neutrophil and macrophage recruitment [42]. Neutrophils contribute to NETosis and release ROS, which amplifies the inflammatory response by further activating the NF-κB pathway. Macrophages further release proinflammatory cytokines, prolonging the inflammatory response and contributing to chronic inflammation. This creates a positive feedback loop of inflammation, NETosis, and platelet hyperactivation that leads to hypercoagulability.

Additionally, MNPs may entrap circulating VWF [43]. Under normal conditions, VWF circulates as a folded protein, and its binding site for GPIbα on the A1 domain is hidden. However, upon vascular injury, VWF unfolds and binds through its collagen-binding sites (A1 and A3 domains) to the exposed subendothelial matrix. Immobilization of VWF to exposed collagen activates the VWF A1 domain, enabling GPIbα binding. The reversible nature of the VWF A1-GPIbα interaction causes platelets to decelerate on immobilized VWF, resulting in adhesion of platelets to the exposed subendothelial matrix. GPIbα-VWF and platelet GPVI-collagen interactions trigger the activation of α_IIb_β_3_ receptors on the platelet surface, as depicted in Figure 3. Initially, α_IIb_β_3_ receptors bind fibrinogen. Plastic particles may adsorb negatively charged proteins, which favors factor (F)XII binding [44]. FXII is then activated to FXIIa, triggering the intrinsic coagulation cascade and increasing thrombin production. Thrombin converts fibrinogen to fibrin, which binds to the α_IIb_β_3_ receptors in the later stages of aggregation. As these α_IIb_β_3_-fibrin interactions increase, platelets aggregate and form platelet plugs within the vascular wall. Excessive platelet aggregation is a key contributor to thrombus formation.

**Figure 3:**
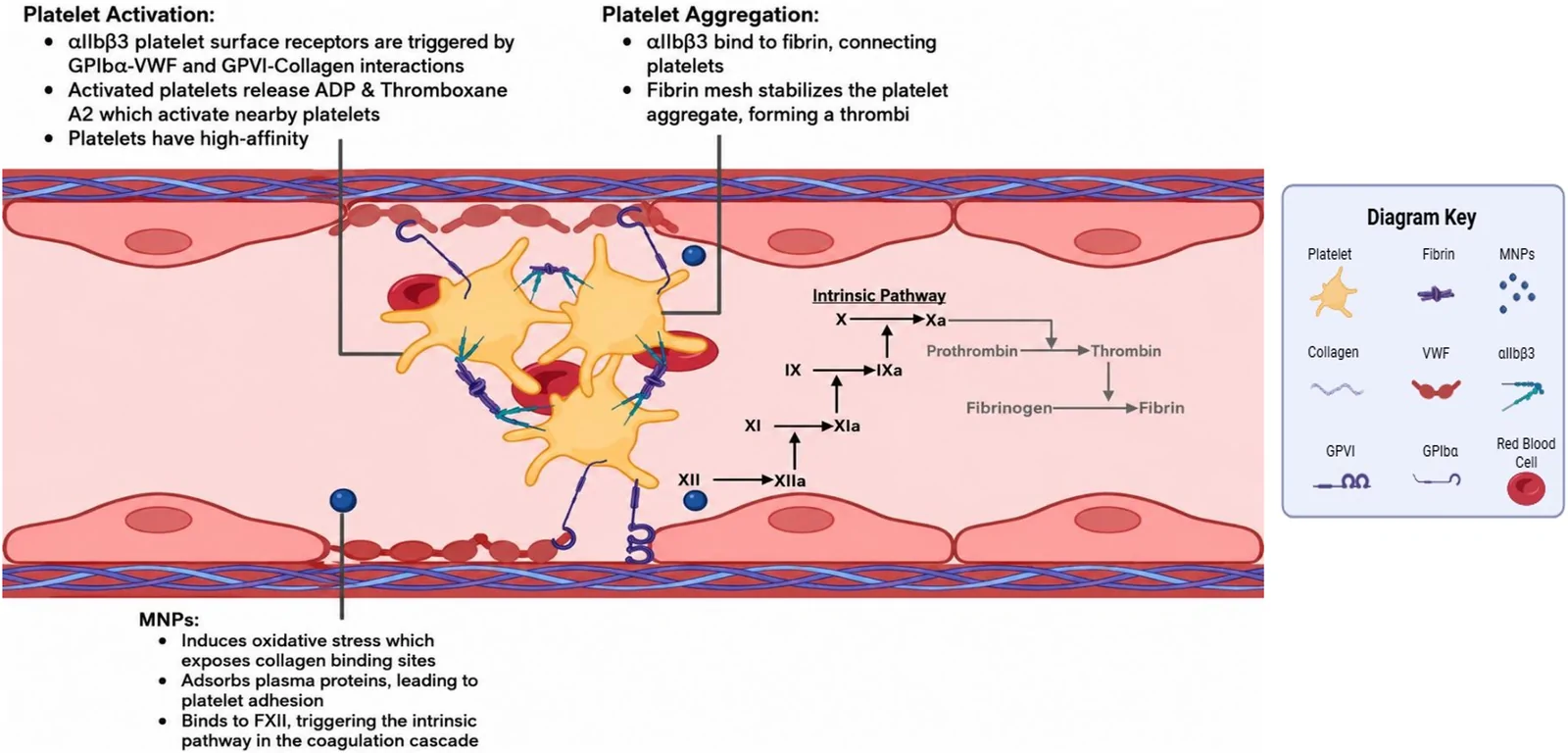
Micro- and nanoplastic (MNP)-mediated platelet activation and contact pathway-driven thrombus formation. MNPs may adsorb plasma proteins, including factor (F)XII, promoting contact pathway activation and thrombin generation. Concurrent endothelial injury and exposure to collagen-binding sites facilitate von Willebrand factor (VWF)- and glycoprotein (GP) VI-mediated platelet adhesion, platelet activation, fibrin formation, and platelet aggregate stabilization. ADP, adenosine diphosphate.

### 3.3 Evidence of MNPs in human blood

Lee et al. [45] found significant linear associations between MP particle counts and high-sensitivity C-reactive protein (CRP) levels, as well as between MP particle counts and fibrinogen. One possible mechanism by which this association may translate into thrombogenic risk is via monomeric CRP, a dissociated form of high-sensitivity CRP [46]. Molins et al. [46] found that monomeric CRP promoted platelet aggregation, an effect partially mediated through integrin α_IIb_β_3_ and CD36, creating a prothrombotic environment. In a case-crossover study of 707 incident venous thromboembolism cases, CRP (measured in 298 patients) was associated with a 1.8-fold increase in venous thromboembolism risk among those without infection and a 1.6-fold increase among those with infection [47].

Moreover, Leslie et al. [48] detected PE, PS, and PE terephthalate in 77% of 22 donor samples. In a multimodal detection study of 30 patients who underwent thrombectomy, MNPs were identified in 24 participants using pyrolysis-gas chromatography-mass spectrometry [10]. The most common polymer identified was PE (53.6%). Polyamide 66 was also detected at high rates, present 81.3% of thrombi from ischemic stroke and 100% of thrombi from deep vein thrombosis. Critically, higher MP concentrations correlated with increased disease severity. Furthermore, the MP-detected group had a significantly higher D-dimer (8.3 ± 1.5 μg/L) than the MP-undetected group (6.6 ± 0.5 μg/L; *P* < .001), indicating heightened active clotting and thrombus breakdown [10]. However, pyrolysis-gas chromatography-mass spectrometry is susceptible to matrix interference, so the aforementioned results should be interpreted with caution [49].

### 3.4 Surface modifications of MNPs as modulators of Virchow’s triad

The thrombogenic mechanisms described by Virchow’s triad do not operate in isolation. They are amplified by polymer type, surface charge, and the weathering effects of MNPs. Once MNPs enter the bloodstream, they adsorb surrounding proteins and form an outer protein corona, suggesting that cells interact with protein-coated MNPs rather than bare plastic materials. The protein corona comprises a hard corona (tightly bound proteins) and a soft corona (loosely bound proteins that quickly interchange) [50]. Proteins adsorb to MNP surfaces due to differences in electrostatic forces, masking the originally hydrophobic NP surface and exposing functional groups. These surface modifications redefine the biological identity of MNPs (Figure 4). MNPs with varying binding profiles have different protein corona compositions and effects on surrounding cells [51,52].

**Figure 4:**
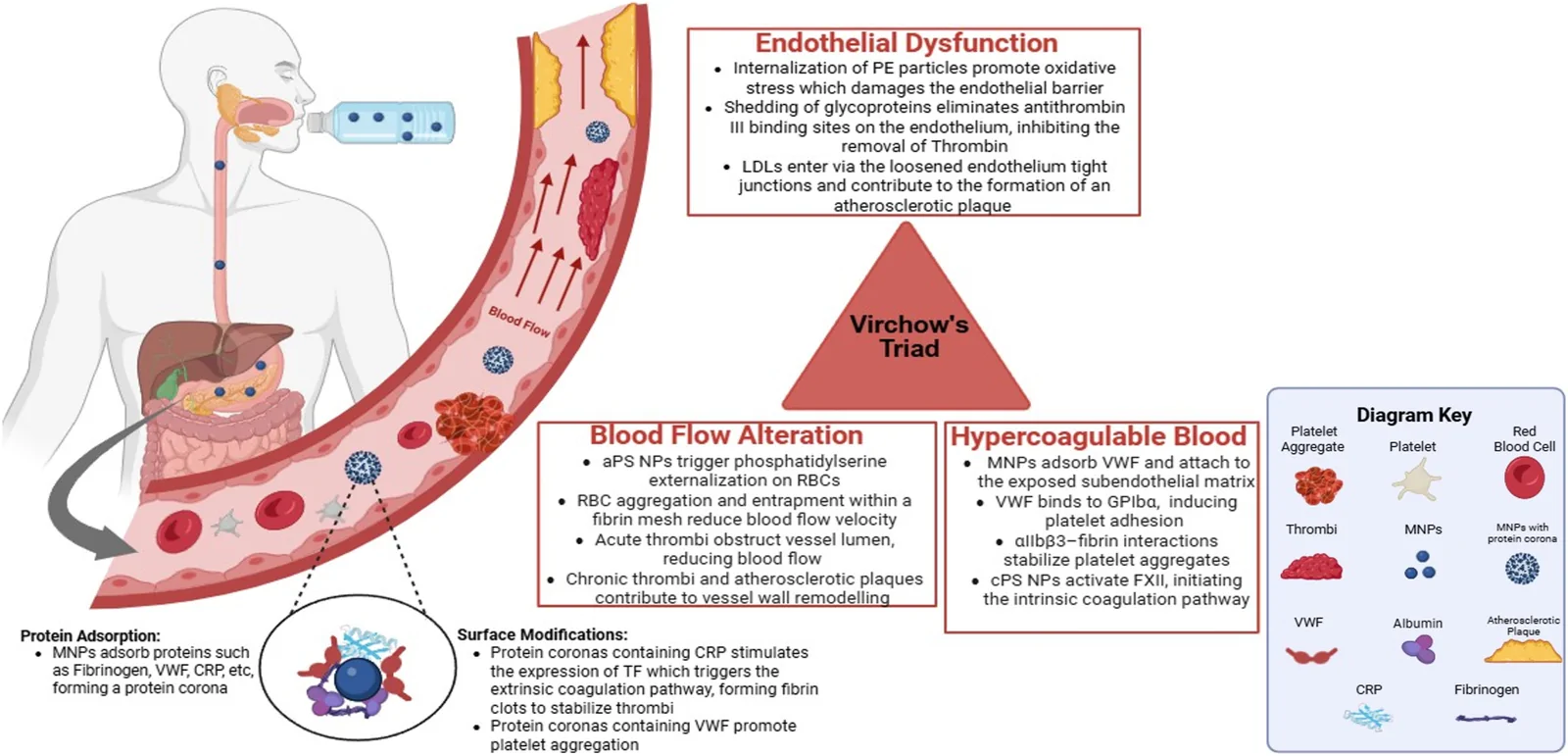
Surface-modified micro- and nanoplastics (MNPs) as modulators of Virchow’s triad in thrombogenesis. MNPs may undergo surface modification and adsorb plasma proteins, forming a protein corona. These altered biological interfaces may promote endothelial dysfunction, altered blood flow, and hypercoagulability through oxidative stress, endothelial barrier disruption, platelet adhesion, red blood cell (RBC) phosphatidylserine externalization, von Willebrand factor (VWF) binding, factor (F)XII activation, and fibrin-rich thrombus stabilization. aPS, aminated polystyrene; cPS, carboxylated polystyrene; CRP, C-reactive protein; GPIbα, glycoprotein Ib platelet subunit α; LDL, low-density lipoprotein; NP, nanoplastic; PE, polyethylene; TF, tissue factor.

### 3.5 Thrombotic effects of polymer type and surface charge of MNPs

While all MNPs pose a thrombotic risk, the specific mechanisms vary significantly by polymer type and surface charge. In a study of 17 atherosclerotic plaques from human arterial samples, pyrolysis-gas chromatography-mass spectrometry identified PE as the most common polymer type, followed by polypropylene (PP), PVC, and PS [53]. PE and PVC particles carry a negative charge, while PS particles have different surface modifications and can carry positive or negative charges, and PP has no charge.

PP MNPs primarily engage through endothelial damage, triggering an inflammatory response via tumor necrosis factor-α, IL-6, and the NLRP3 inflammasome, which damages the vascular endothelial wall by suppressing the protein Zonula Occludens-1 [54]. Zonula Occludens-1 functions as a scaffolding protein that links tight junction proteins to the underlying actin cytoskeleton. Together, these tight junctions form a selectively permeable barrier that regulates the passage of substances through the intercellular space [55]. Depletion of intercellular junctions may compromise vascular barrier integrity, potentially allowing lipids and immune cells to enter the arterial wall. Sun et al. [56] found that in rats, atheroma formation may result from loss of tight junction proteins and resulting permeability. This promotes atherosclerosis, a pathological sequela of endothelial damage [54].

Similarly, PE and PVC further perpetuate endothelial damage through distinct mechanisms. Once PE particles are internalized by macrophages, they disrupt mitochondrial function by interfering with the electron transport chain, leading to excessive ROS generation and damage to the endothelial lining. This subsequently triggers the extrinsic coagulation pathway [57]. The acquired protein corona presents surface epitopes that leukocyte pattern recognition receptors may bind, activating the NLRP3 inflammasome and NF-κB signaling pathway and releasing proinflammatory cytokines [58].

Alternatively, PS MNPs may induce hypercoagulability or alter blood flow. As demonstrated by Nemmar et al. [59], positively charged ultrafine plastic particles significantly increase thrombosis rates, whereas negatively charged and unmodified ultrafine particles of the same size do not. Negatively charged cPS NPs may initiate the intrinsic coagulation pathway by activating FXII [60,61], leading to hypercoagulability. In contrast, positively charged aPS NPs bind to the negatively charged red blood cell (RBC) membrane and disrupt the lipid bilayer. This externalizes phosphatidylserine residues, increasing RBC adhesion to other RBCs and the endothelial lining [62]. Moreover, phosphatidylserine externalization activates tissue factor production, which activates the extrinsic coagulation cascade and enhances thrombin generation [63]. As RBCs accumulate within the fibrin mesh, flow is obstructed, and velocity is reduced. Furthermore, RBCs trapped within the fibrin mesh form larger thrombi that directly obstruct blood flow. Table 1 summarizes the mechanisms of various polymer types that contribute to each pillar of Virchow’s triad. In summary, much of the mechanistic evidence comes from PS NPs, which are useful laboratory models but may not represent the broader human MNP exposome. Human thrombus and atheroma studies more commonly detect PE and PVC particles. Therefore, PS NP findings should be interpreted as proof-of-principle evidence rather than generalized to all MNPs.

**Table 1:** Thrombotic mechanisms of different plastic polymer types and surface charges

Plastic polymer	Surface charge	Coagulation pathway	Key mechanism	Pillar of Virchow’s triad	Reference
PE	Negative	Extrinsic	Internalized by macrophages to elevate ROS production	Endothelial damage	Koner et al. [57]
PP	Uncharged	Structural contribution	Induces inflammatory responses that destroy tight junctions, facilitating atherogenesis	Endothelial damage	Wang et al. [54]
PVC	Negative	Extrinsic	Interaction with leukocyte receptor elevates ROS production	Endothelial damage	Yu et al. [58]
PS	Carboxylated (negative)	Intrinsic	Activation of FXII	Hypercoagulability	Oslakovic et al. [61]
	Aminated (positive)	Extrinsic	Activates the production of TF and causes RBC adhesion	Hypercoagulability, blood flow alteration	Argañaraz et al. [63]

FXII, factor XII; PCV, polyvinyl chloride; PE, polyethylene; PP, polypropylene; PS, polystyrene; RBC, red blood cell; ROS, reactive oxygen species; TF, tissue factor.

### 3.6 Weathering effect of MNPs

Weathered MNPs (which transform inert plastics into highly reactive particles) may induce hypercoagulability. Exposing hydrophobic pristine MNPs to UV light or fluctuating temperatures photo-oxidizes their polymer chains, breaking carbon–carbon bonds and introducing oxygen-containing functional groups [64]. As a result, weathered MNPs interact with complex proteins and develop a hard protein corona. Surface oxidation on weathered particles adsorbs coagulation factors such as fibrinogen and FXII. Moreover, submicron weathered particles penetrate deeper into the bloodstream and trigger platelet activation at much lower concentrations than pristine particles [65]. Therefore, by altering the balance between coagulation factors and activating platelets, weathered MNPs may induce hypercoagulability.

### 3.7 Evidence of surface-modified MNPs influencing thrombogenesis

Kim et al. [62] demonstrated that RBCs exposed to aPS NPs exhibited enhanced procoagulant activity and promoted greater thrombin generation than those exposed to cPS NPs and nPS NPs. They also concluded that 100 nm (A100) aPS NPs caused the most potent hemolysis of human RBCs. Interestingly, 50 nm (A50) aPS NPs, which have a larger surface area than A100 aPS NPs at the same concentration, were less effective than A100 aPS NPs. This suggests that surface modification, size, and surface area are important determinants of MNP-induced toxicity. The rate of hemolysis was ∼5% for A50 aPS NPs, 40% for A100 aPS NPs, ∼5% for 1000 nm aPS NPs, ∼2% for A100 cPS NPs, and ∼3% for A100 nPS NPs. These findings indicate that aPS NPs have a greater impact on hemolysis than cPS NPs and nPS NPs [62]. Observations of carbon nanotubes further highlight the thrombogenic role of polymer coatings. Carboxylated, multiwalled carbon nanotubes coated with PE glycol were more thrombogenic than pluronic-coated, carboxylated, multiwalled carbon nanotubes at all concentrations tested (10, 50, and 100 μg/mL) [66].

Ferreira et al. [67] correlated elevated maximum amplitude (MA) in thromboelastography with thrombosis, which may indicate hypercoagulability. Christodoulides et al. [39] found that at 250 μg/mL, A50 cPS NPs yielded an MA of 46 mm and an R-time of 14 minutes; A100 cPS NPs yielded an MA of ∼70 mm and an R-time of 6 minutes; and 500 nm cPS NPs yielded an MA of ∼70 mm and an R-time of 6 minutes. These findings suggest that the procoagulant effects of cPS NPs are related to size. A potential reason 500 nm cPS NPs have a lower MA than A100 cPS NPs may be due to differences in protein corona composition. Different compositions can affect how these particles interact with platelet receptors. At 250 μg/mL, the MA of nPS NPs was slightly lower: A50 particles had an MA of 45 mm and an R-time of 18 minutes; A100 particles had an MA of 60 mm and an R-time of 13 minutes; and 500 nm particles had an MA of 55 mm and an R-time of 14 minutes. At a concentration of 250 μg/mL, the MA of aPS NPs was 70 mm for A100 particles and 55 mm for 500 nm particles. The R-time was 5 minutes for A100 particles and ∼14 minutes for 500 nm particles. Table 2 presents only 1 polymer type to compare direct links between surface modifications, size, and thrombosis.

**Table 2:** Comparison of surface-modified polystyrene nanoplastics and their thrombotic effects

Surface modification of PS NPs	Size (nm)	TEG		Hemolytic rate (%)	Biggest contributor to thrombosis	Reference
		MA (mm; normal: 50-70*)*	R-time (min; normal: 7.5-15)			
nPS	50	**45**	**18**	-	100 nm NPs	Christodoulides et al. [39]
	100	60	∼13	∼3		Kim et al. [62]
	500	55	∼14	-		Christodoulides et al. [39]
aPS	50	-	-	∼5	100 nm NPs	Kim et al. [62]
	100	70	**5**	40		Christodoulides et al. [39] and Kim et al. [62]
	500	55	∼14	-		Christodoulides et al. [39]
	1000	-	-	∼5		Kim et al. [62]
cPS	50	**46**	14	-	500 nm NPs	Christodoulides et al. [39]
	100	∼68	**6**	∼2		Christodoulides et al. [39] and Kim et al. [62]
	500	∼70	**6**	-		Christodoulides et al. [39]

Bold font indicates abnormal values.

aPS, aminated polystyrene; cPS, carboxylated polystyrene; MA, maximum amplitude; NP, nanoplastic; nPS, nonfunctionalized polystyrene; PS, polystyrene; TEG, thromboelastography.

Based on the results above, we postulate that surface modification is the primary determinant of prothrombotic effects, with aPS NPs showing the largest effect. Particle size is a secondary determinant. This suggests an optimal size range for certain polymers to have a higher potential prothrombotic effect. However, the concentrations used in experimental studies also require cautious interpretation. For example, thromboelastography effects reported at 250 μg/mL exceed many reported human blood MNP concentrations, which are typically in the μg/L range. This exposure gap highlights the need for dose-response studies using clinically and environmentally relevant concentrations.

## 4 Discussion

This review synthesizes emerging evidence that may position MNPs as a prothrombotic factor, with potential effects mapped onto Virchow’s triad. These mechanisms may be influenced by polymer type, surface charge, and the weathering effects of MNPs. For instance, inflammatory responses induced by PP particles may suppress the tight junction protein Zonula Occludens-1, leading to lipid and monocyte infiltration into the intima and facilitating plaque formation [54]. Prior studies [39,62] suggest that A100 aPS NPs have the highest MA and the greatest hemolytic rate and, consequently, the largest prothrombotic effect. Additionally, submicron weathered particles penetrate deeper into the bloodstream and may trigger platelet activation at much lower concentrations than pristine particles [65]. With greater surface roughness, oxygen-containing functional groups, and protein adsorption, weathered MNPs may induce more platelet activation and coagulation and may therefore be more biologically relevant than pristine laboratory particles. Future studies should compare pristine and weathered particles across clinically relevant dose ranges.

Endothelial damage is a critical component of Virchow’s triad that links MNP exposure to thrombogenesis. MNP-induced oxidative stress compromises the endothelial barrier and causes GP shedding [23]. When GPs are shed, less thrombin is sequestered from the bloodstream, and the resulting excess contributes to thrombus formation. Additionally, experimental studies indicate that shedding of glycocalyx chains alters near-wall blood flow velocity and increases blood flow disorder [34]. Endothelial cell damage loosens tight junctions, leading to intimal LDL accumulation and plaque formation [25]. Human studies have shown associations between MNP particle counts and elevated levels of high-sensitivity CRP and fibrinogen [45]. Moreover, monomeric CRP significantly induces platelet aggregation, which is partially mediated by integrin α_IIb_β_3_ and CD36 [46].

Despite advances in understanding how MNP exposure links to thrombogenesis, several limitations warrant consideration. Most evidence comes from *in vitro* or animal models and may not directly reflect human pathophysiology. Current studies may not be sufficiently comprehensive to determine the threshold concentration at which MNPs pose a severe thrombotic risk. Moreover, the lack of standardized methods for detecting MNPs in biological samples introduces interstudy variability across the entire analytical workflow. For example, nitrile and latex laboratory gloves shed stearate particles into laboratory samples, potentially resulting in an overestimation of ∼2000 false-positive particles/mm^2^ under standard handling conditions [68]. Future studies could compare pristine MNPs with glove-derived stearates using clean-air handling and rigorous procedural blanks to determine whether MNP-induced inflammation is a chemical effect masquerading as a particulate effect.

MNP characterization is also imperfect, as pyrolysis-gas chromatography-mass spectrometry is susceptible to matrix interference. For instance, lipids thermally decompose into the same products as PE, generating false positives [49]. Rauert et al. [49] addressed this challenge by operating pyrolysis-gas chromatography-mass spectrometry in double-shot mode and adding a thermal desorption step at 300 °C before pyrolysis at 650 °C, reducing background signal and improving detection limits. Despite these advances, multiple identification techniques should be combined to cross-validate the identity of plastic particles. International standards, such as International Organization for Standardization 24187:2023, are essential for differentiating true MNPs from contamination introduced during sampling, sample preparation, or detection [69]. Finally, the presence of confounding variables, such as chronic inflammation, hinders delineation of potential thrombotic effects from MNP exposure. The concentration at which MNPs may pose a serious thrombotic risk, and whether this is cumulative or triggered by high concentrations, remains unclear. Therefore, future research must prioritize standardized, high-quality methods and long-term studies to corroborate the potential dose-dependent relationship between MNP exposure and thrombogenesis.

Current antithrombotic protocols rely heavily on anticoagulants such as vitamin K antagonists, heparin, or direct oral anticoagulants [70]. However, the mechanisms identified in this review suggest that MNP-induced thrombogenesis may be resistant to these standard interventions. For instance, current protocols do not address the mechanical obstruction caused by MNPs engulfed by macrophages. Emerging technologies, such as ultrasound-mediated thrombolysis, microrobots, electrothrombectomy, and magnetic nanoparticles, may introduce potential future solutions [71]. Moreover, not all anticoagulants reduce ROS levels, and they may not prevent subsequent plaque rupture and thrombus formation. De Iorio et al. [72] found significantly lower ROS levels in myeloid cells in lithium heparin and acid citrate dextrose formula A than in tripotassium ethylenediaminetetraacetic acid samples. As such, while traditional risk factors primarily drive thrombosis, assessing biomarkers specific to MNP exposure, such as ROS levels or thrombus composition, enables more personalized treatment plans for administering anticoagulants to high-risk patients. A further limitation is that many mechanistic studies use pristine or surface-functionalized PS NPs at concentrations higher than those typically measured in human blood. These models are useful for defining pathways but should not be generalized uncritically to weathered environmental particles or clinically prevalent polymers such as PE and PVC.

## 5 Conclusion

Detecting MNPs in circulating blood raises concerns about their systemic availability and long-term implications. The current literature suggests that MNPs may be associated with thrombogenesis through key thrombotic mechanisms that influence Virchow’s triad. However, current conclusions are constrained by reliance on *in vitro* or animal models, confounding factors, and nonstandardized measurement methods, highlighting areas for future research to better evaluate the potential role of MNPs in thrombogenesis.
