# Supplementary material for: Microplastics, nanoplastics, and thrombogenesis
Source: Res Pract Thromb Haemost. 2026 Aug 28;10(7):106931. doi: 10.1016/j.rpth.2026.106931 (PMC13677222; doi:10.1016/j.rpth.2026.106931)
Supplement: Supplementary file 1 [file mmc1.docx]

**Appendix**

**Search Strategy**………………………………………………………………………………………………………………..Page 1

**References Listed in the:**

Introduction……………………….……………………………………………………………………………………………Page 4

Cellular Uptake of MNPs………………………….…..………………………………………………………………….Page 7

Virchow’s Triad………………………………………………………………………………………………………………..Page 10

Evidence of MNPs in Human Blood……...………….…………….………………………………………………..Page 14

Surface Modifications of MNPs as modulators of Virchow’s Triad……………..…………………….Page 15

Others……………………………………………………………………………………………………………..……………...Page 21

**Search Strategy**

**PubMed**

| **No.** | **Query** | **Result** |
| --- | --- | --- |
| 1 | Microplastic [MeSH] OR Microplastic [Title/Abstract] OR Nanoplastic [MeSH] OR Nanoplastic [Title/Abstract] OR plastic particle [Title/Abstract] OR polymer particle [Title/Abstract] OR secondary microplastic [Title/Abstract] OR primary microplastic [Title/Abstract] OR micro polymer [Title/Abstract] OR nano polymer [Title/Abstract] | 25,059 |
| 2 | Embol* [Title/Abstract] OR thrombosis [MeSH] OR thrombosis [Title/Abstract] OR thrombus [Title/Abstract] OR thrombi [Title/Abstract] OR thrombotic [Title/Abstract] OR thromboembol* [Title/Abstract] | 505,172 |
| 3 | #1 AND #2 | 55 |

**Embase**

| **No.** | **Query** | **Result** |
| --- | --- | --- |
| 1 | ‘Microplastics’/exp OR ‘Nanoplastic’/exp OR ‘Microplastic’:ti,ab OR ‘Nano plastic’:ti,ab OR ‘plastic particle’:ti,ab OR ‘polymer particle’:ti,ab OR ‘secondary microplastic’:ti,ab OR ‘primary microplastic’:ti,ab OR ‘micro polymer’:ti,ab OR ‘nano polymer’:ti,ab | 21,373 |
| 2 | Embol*:ti,ab OR thrombosis:ti,ab OR thrombus:ti,ab OR thrombi:ti,ab OR thrombotic:ti,ab OR thromboembol*:ti,ab | 693,649 |
| 3 | #1 AND #2 | 49 |

**Scopus**

| **No.** | **Query** | **Result** |
| --- | --- | --- |
| 1 | TITLE-ABS (Microplastic OR Nanoplastic OR Nano plastic OR plastic particle OR polymer particle OR secondary microplastic OR primary microplastic OR micro polymer OR nano polymer) | 2,055 |
| 2 | TITLE-ABS (embol* OR thrombosis OR thrombus OR thrombi OR thrombotic OR thromboembol*) | 62,954 |
| 3 | #1 AND #2 | 877 |

| **Category** | **No of articles** |
| --- | --- |
| Introduction | 10 |
| Cellular Uptake of MNPs | 10 |
| Virchow’s Triad | 21 |
| Evidence of MNPs in Human Blood | 2 |
| Surface Modifications of MNPs as modulators of Virchow’s Triad | 14 |
| Others | 2 |
| **Total** | **64** |

**Introduction**

| **No.** | **Author** | **Title** | **Objective** | **Summary** |
| --- | --- | --- | --- | --- |
| 1. | Landrigan PJ et al. (2025) | The Lancet Countdown on health and plastics | Provide a comprehensive overview of the negative impact that plastics pose to humans and the planet | This study mentions that plastics pose grave threats to human and planetary health, with production growing from 2 megatonnes in 1950 to 475 megatonnes in 2022. Plastic chemicals and MNPs are linked to cardiovascular disease, cancer, and reproductive harm. The authors argue harms are preventable and announce the Lancet Countdown on Health and Plastics to track progress under the Global Plastics Treaty. |
| 2. | Banerjee A et al. (2021) | Micro- and nanoplastic induced cellular toxicity in mammals: A review | Review the cellular toxicity mechanisms of MNPs in mammalian systems | This study aims to synthesize evidence that MNPs induce cellular toxicity in mammals through oxidative stress, inflammation, genotoxicity, and endocrine disruption. In vitro and in vivo studies demonstrate dose- and size-dependent toxic effects across multiple organ systems. The review highlights significant gaps in knowledge and calls for standardized toxicity testing. |
| 3. | Winiarska E et al. (2024) | The potential impact of nano- and microplastics on human health: Understanding human health risks | To evaluate the potential impact of nano- and microplastics on human health, focusing on exposure routes and associated health risks. | This study mentions that MNPs enter the human body via ingestion, inhalation, and dermal routes, accumulating in multiple tissues. They trigger inflammatory responses, oxidative stress, and endocrine disruption. The authors emphasize the need for greater understanding of exposure thresholds and long-term health consequences. |
| 4. | Menichetti A et al. (2025) | Penetration of Microplastics and Nanoparticles Through Skin: Effects of Size, Shape, and Surface Chemistry | To investigate the penetration of microplastics and nanoparticles through skin, examining how size, shape, and surface chemistry influence dermal uptake. | This study discusses how particles smaller than 100 nm can penetrate the stratum corneum, with surface chemistry and shape significantly modulating penetration depth. Smaller, charged, and irregularly shaped particles demonstrate greater skin penetration. The findings highlight dermal exposure as an underappreciated route for MNP entry into the body. |
| 5. | Cao G et al. (2023) | Getting Health Hazards of Inhaled Nano/Microplastics into Focus: Expectations and Challenges | To bring into focus the health hazards of inhaled nano/microplastics, discussing current evidence, expectations, and analytical challenges. | This study mentions that inhaled MNPs deposit in the respiratory tract and can translocate to systemic circulation. They induce pulmonary inflammation, oxidative stress, and may contribute to cardiovascular and neurological harm. The authors highlight significant analytical challenges in quantifying inhalation exposure. |
| 6. | Ketenci HÇ et al. (2026) | Iatrogenic Microplastic Exposure: A Possible and Underrecognized Healthcare-Associated Exposure Framework in Human Medicotoxicological Risk | To propose and characterise iatrogenic microplastic exposure as a possible and underrecognised healthcare-associated exposure framework contributing to human medicotoxicological risk. | This study raises awareness on how medical procedures including intravenous infusions, blood transfusions, and implantation of polymer-containing devices represent significant but underappreciated sources of MNP exposure. The authors propose a framework for assessing iatrogenic MNP risk and call for regulatory attention to plastic use in healthcare settings. |
| 7. | Cox KD et al. (2019) | Human Consumption of Microplastics | Quantify human dietary microplastic consumption through food and beverages | Estimates that adults consume 74,000 to 121,000 microplastic particles per year through food and beverages, with shellfish, tap and bottled water, and salt among the highest-contributing sources. |
| 8. | Vanetti C et al. (2025) | Effects of microplastics on the immune system: How much should we worry? | To review the effects of microplastics on the immune system, assessing the extent to which current evidence warrants concern | This study points out that MNPs activate innate immune responses, promote macrophage-mediated inflammation, and dysregulate cytokine production. They can impair adaptive immunity and trigger allergic and autoimmune responses in experimental models. The authors conclude that immune effects warrant serious concern given widespread human exposure. |
| 9. | Zhang T et al. (2025) | Tiny trouble: microplastics, nanoplastics, and their heartfelt impact on cardiovascular health | To review the cardiovascular health impacts of microplastics and nanoplastics, focusing on mechanisms linking MNP exposure to cardiac and vascular pathology. | This study mentions that MNPs promote cardiovascular disease through endothelial dysfunction, oxidative stress, inflammation, platelet activation, and coagulation cascade disruption. They have been detected in atherosclerotic plaques, thrombi, and cardiac tissue. The review identifies MNPs as emerging cardiovascular risk factors requiring urgent clinical and regulatory attention. |
| 10. | Wang T et al. (2024) | Multimodal detection and analysis of microplastics in human thrombi from multiple anatomically distinct sites | To detect and characterize microplastics in human thrombi collected from multiple anatomically distinct sites using multimodal detection methods | This study detects microplastics in thrombi from arterial, venous, and cardiac sites in human patients, with polystyrene and polyethylene being the most prevalent polymers. Particle burden correlated with inflammatory markers in some sites. The findings directly implicate MNPs in human thrombogenesis across multiple vascular territories. |

**Cellular Uptake of Micro and Nano plastics**

| **No.** | **Author** | **Title** | **Objective** | **Summary** |
| --- | --- | --- | --- | --- |
| 11. | Lett Z et al. (2021) | Environmental microplastic and nanoplastic: Exposure routes and effects on coagulation and the cardiovascular system | To review the exposure routes of environmental MNPs and their effects on coagulation and the cardiovascular system. | This study mentions that MNPs enter the body via ingestion, inhalation, and dermal routes, reaching systemic circulation and disrupting coagulation through platelet activation, fibrinogen adsorption, and endothelial damage. Surface charge and polymer type modulate prothrombotic potential. The review calls for further human studies to clarify thrombotic risk thresholds. |
| 12. | Zhu X et al. (2023) | Micro- and nanoplastics: A new cardiovascular risk factor? | To evaluate micro- and nanoplastics as potential new cardiovascular risk factors, reviewing evidence from experimental and epidemiological studies. | This study discusses how MNPs accumulate in cardiovascular tissues and promote atherosclerosis, endothelial dysfunction, and thrombosis through inflammatory and oxidative mechanisms. Emerging human data including detection of MNPs in atheromas support their role as cardiovascular risk factors. The authors call for inclusion of MNP exposure in cardiovascular risk assessment frameworks. |
| 13. | Aguilar-Guzmán JC et al. (2022) | Polyethylene terephthalate nanoparticles effect on RAW 264.7 macrophage cells | To investigate the effects of polyethylene terephthalate nanoparticles on RAW 264.7 macrophage cells, focusing on cytotoxicity and inflammatory responses. | This study mentions that polyethylene terephthalate nanoparticles induced dose-dependent cytotoxicity, reactive oxygen species production, and pro-inflammatory cytokine release in macrophages. Particle internalization was confirmed and associated with lysosomal disruption. The findings suggest polyethylene terephthalate nanoparticles pose significant inflammatory risk upon cellular uptake. |
| 14. | Liu L et al. (2021) | Cellular internalization and release of polystyrene microplastics and nanoplastics | To investigate the cellular internalisation and release of polystyrene microplastics and nanoplastics, examining size-dependent uptake mechanisms. | This study demonstrates that polystyrene MNPs are internalized by cells via endocytosis in a size-dependent manner, with smaller particles showing greater uptake efficiency. Particles are retained intracellularly for extended periods and can be released, potentially spreading to adjacent cells. The study highlights the potential for intracellular accumulation and chronic cellular toxicity. |
| 15. | Sousa de Almeida M et al. (2021) | Understanding nanoparticle endocytosis to improve targeting strategies in nanomedicine | To review nanoparticle endocytosis pathways and how understanding these mechanisms can improve targeting strategies in nanomedicine. | This study illustrates that nanoparticles are internalized through multiple endocytic pathways including clathrin-mediated endocytosis, macropinocytosis, and caveolae-mediated uptake, with pathway selection influenced by particle size, shape, and surface chemistry. Protein corona formation significantly alters cellular interactions. Understanding these mechanisms is critical for designing effective drug delivery systems and for predicting MNP toxicity. |
| 16. | Geppner L et al. (2025) | Effects of micro- and nanoplastics on blood cells in vitro and cardiovascular parameters in vivo, considering their presence in the human bloodstream and potential impact on blood pressure | To assess the effects of micro- and nanoplastics on blood cells in vitro and cardiovascular parameters in vivo, considering their presence in the human bloodstream. | This study shows that MNPs induce hemolysis, platelet activation, and alterations in blood cell morphology in vitro, with cardiovascular effects including blood pressure changes observed in vivo. Effects were size- and concentration-dependent. The findings support MNPs as clinically relevant cardiovascular stressors present in human blood. |
| 17. | Vlacil AK et al. (2021) | Polystyrene microplastic particles induce endothelial activation | To investigate whether polystyrene microplastic particles induce endothelial activation, a key early step in vascular inflammation and atherosclerosis. | This study demonstrates that polystyrene microplastics induces endothelial cell activation characterized by upregulation of adhesion molecules promoting leukocyte adhesion. NF-κB signalling was identified as a key mediator of this response. The findings implicate microplastic exposure in early-stage vascular inflammation. |
| 18. | Giustarini G et al. (2025) | Polystyrene microplastics cross the murine intestine and induce inflammatory cell death after phagocytosis by human monocytes and neutrophil | To investigate whether polystyrene microplastics can cross the murine intestine and induce inflammatory cell death following phagocytosis by human monocytes and neutrophils. | This study explains that polystyrene microplastics crossed the intestinal barrier in murine models and were phagocytosed by human monocytes and neutrophils, inducing inflammatory cell death. This was associated with NLRP3 inflammasome activation and IL-1β release. The findings demonstrate a direct link between MNP intestinal translocation and systemic innate immune activation. |
| 19. | Thapliyal C et al. (2025) | Mechanistic insight into potential toxic effects of microplastics and nanoplastics on human health | To provide mechanistic insight into the potential toxic effects of microplastics and nanoplastics on human health across multiple organ systems. | This study elaborates on how MNPs exert toxicity through oxidative stress, inflammatory pathway activation including NF-κB and mitochondrial dysfunction, and endocrine disruption. They affect the respiratory, cardiovascular, reproductive, and nervous systems. The review emphasizes that polymer type, particle size, and surface modification are key determinants of toxicity magnitude. |
| 20. | Huang H et al. (2025) | Microplastics in the bloodstream can induce cerebral thrombosis by causing cell obstruction and lead to neurobehavioral abnormalities | To investigate whether microplastics in the bloodstream can induce cerebral thrombosis through cell obstruction and to assess the resulting neurobehavioral consequences. | This study ascertains the effects of microplastics in the bloodstream causing physical obstruction of cerebral microvessels, leading to thrombosis and ischaemic injury in murine models. Affected animals exhibited significant neurobehavioral abnormalities including cognitive and motor deficits. The findings provide direct experimental evidence linking circulating microplastics to cerebrovascular thrombosis. |

**Virchow’s Triad**

| **No.** | **Author** | **Title** | **Objective** | **Summary** |
| --- | --- | --- | --- | --- |
| 21. | Wei W et al. (2022) | Anionic nanoplastic exposure induces endothelial leakiness | To investigate whether anionic nanoplastic exposure induces endothelial leakiness and to characterize the underlying molecular mechanisms. | This study outlines how anionic nanoplastics induced endothelial leakiness by disrupting vascular endothelial-cadherin junction integrity, increasing vascular permeability. Effects were charge-dependent, with anionic particles causing greater disruption than neutral or cationic particles. The findings highlight surface charge as a critical determinant of nanoplastic endothelial toxicity. |
| 22. | Sivakumar R et al. (2025) | From Environment to Endothelium: The Role of Microplastics in Vascular Aging. Microplastics | To review the role of microplastics in vascular ageing, examining how MNP exposure from the environment to the endothelium contributes to accelerated vascular senescence. | This study demonstrates that MNPs promote endothelial senescence through oxidative stress, telomere shortening, inflammatory cytokine release, and impairment of endothelial repair mechanisms. These processes accelerate vascular ageing and increase susceptibility to atherosclerosis and thrombosis. The review calls for MNP exposure to be incorporated into models of vascular ageing and cardiovascular risk. |
| 23. | Mahmud F et al. (2024) | Molecular and Cellular Effects of Microplastics and Nanoplastics: Focus on Inflammation and Senescence | To examine the molecular and cellular effects of microplastics and nanoplastics with a focus on inflammation and cellular senescence. | This study discusses how MNPs trigger chronic inflammation through pattern recognition receptor activation, inflammasome assembly, and cytokine release, while simultaneously inducing cellular senescence via DNA damage responses and mitochondrial dysfunction. These two processes reinforce each other, creating a self-amplifying cycle of tissue damage. The authors propose that MNP-induced inflammaging is a key mechanism underlying their long-term health harms. |
| 24. | Lin S et al. (2022) | Metabolomics Reveal Nanoplastic-Induced Mitochondrial Damage in Human Liver and Lung Cells | To use metabolomics to reveal how nanoplastic exposure induces mitochondrial damage in human liver and lung cells. | This study illustrates that nanoplastic exposure caused significant mitochondrial dysfunction in human hepatocyte and lung cell lines. Metabolomic profiling revealed dose-dependent metabolic reprogramming consistent with mitochondrial stress. The findings provide mechanistic insight into the organotoxicity of nanoplastics at the cellular level. |
| 25. | Marfella R et al. (2024) | Microplastics and Nanoplastics in Atheromas and Cardiovascular Events | To investigate whether microplastics and nanoplastics are present in human atheromas and whether their presence is associated with adverse cardiovascular events. | This study demonstrates that MNPs were detected in carotid endarterectomy specimens from patients with atherosclerosis, and their presence was associated with a significantly higher risk of myocardial infarction, stroke, and death at 34-month follow-up compared to patients without detectable MNPs in plaques. This landmark study provides the first direct human evidence linking MNP accumulation in atherosclerotic tissue to adverse cardiovascular outcomes. |
| 26. | Hwang J et al. (2020) | Potential toxicity of polystyrene microplastic particles | To evaluate the potential toxicity of polystyrene microplastic particles, examining cytotoxicity, oxidative stress, and inflammatory responses in vitro. | This study shows that polystyrene microplastics induced cytotoxicity, reactive oxygen species generation, and inflammatory cytokine release in a size- and concentration-dependent manner. Smaller particles exhibited greater toxicity, and surface modifications influenced cellular responses. The findings support polystyrene microplastics as biologically active particles with significant toxicological potential. |
| 27. | Luo WQ et al. (2025) | Size-Dependent Internalization of Polystyrene Microplastics as a Key Factor in Macrophages and Systemic Toxicity | To investigate size-dependent internalization of polystyrene microplastics by macrophages and the resulting systemic toxicity. | Smaller polystyrene microplastic particles were preferentially internalized by macrophages via phagocytosis, triggering greater inflammatory responses and systemic toxicity compared to larger particles. Internalization led to lysosomal damage, NLRP3 inflammasome activation, and IL-1β secretion. Size is confirmed as a key factor in determining macrophage-mediated MNP toxicity. |
| 28. | Hernández AF et al. (2025) | Cellular and Molecular Mechanisms of Micro- and Nanoplastics Driving Adverse Human Health Effects | To review the cellular and molecular mechanisms through which micro- and nanoplastics drive adverse human health effects. | This study illustrates that MNPs exert adverse health effects through direct cellular toxicity, oxidative stress induction, inflammatory pathway activation, endocrine disruption, and epigenetic modifications. They interact with proteins, lipids, and nucleic acids, disrupting fundamental cellular processes across multiple organ systems. The review synthesizes mechanistic evidence supporting regulatory action to limit MNP exposure. |
| 29. | Kaiser M et al. (2026) | Microplastics: A Modifiable Cardiac Risk Factor | To characterize microplastics as a modifiable cardiac risk factor, reviewing evidence for their cardiovascular toxicity and implications for clinical cardiology. | This study describes how microplastics accumulate in cardiac tissue and promote cardiovascular disease through endothelial dysfunction, plaque destabilization, thrombosis, and direct myocardial toxicity. Their modifiable nature through dietary and environmental interventions positions them as a novel target for cardiovascular risk reduction. The authors argue for integration of MNP exposure assessment into cardiac risk stratification. |
| 30. | Christodoulides A et al. (2023) | Exploring microplastic impact on whole blood clotting dynamics utilizing thromboelastography | To explore the impact of microplastics on whole blood clotting dynamics using thromboelastography. | This study illustrates that microplastic exposure significantly altered clotting dynamics as measured by thromboelastography, with anionic polystyrene nanoparticles showing the greatest prothrombotic effect including increased maximum amplitude and clot strength. Effects were surface charge- and size-dependent, with smaller anionic particles exerting the most pronounced procoagulant influence. TEG is proposed as a useful tool for assessing MNP thrombogenicity. |
| 31. | Lunov O et al. (2011) | Amino-Functionalized Polystyrene Nanoparticles Activate the NLRP3 Inflammasome in Human Macrophages | To investigate whether amino-functionalized polystyrene nanoparticles activate the NLRP3 inflammasome in human macrophages. | This study shows that amino-functionalized polystyrene nanoparticles activated the NLRP3 inflammasome in human macrophages, triggering IL-1β secretion through lysosomal destabilization. This inflammatory response was dependent on particle surface chemistry, with amine groups playing a critical role. The findings establish nanoparticle surface functionalization as a key determinant of inflammasome-mediated toxicity. |
| 32. | Chen J et al. (2023) | Surface functionalization-dependent inflammatory potential of polystyrene nanoplastics through the activation of MAPK/ NF-κB signaling pathways in macrophage Raw 264.7 | To investigate the surface functionalisation-dependent inflammatory potential of polystyrene nanoplastics through MAPK/NF-κB signalling pathway activation in macrophages. | This study demonstrates that surface-modified polystyrene nanoplastics activated MAPK/NF-κB signalling pathways in RAW 264.7 macrophages in a functionalization-dependent manner, with carboxylated particles inducing greater inflammatory responses than unmodified particles. Downstream cytokine production including Tumour Necrosis Factor-alpha and IL-6 was significantly upregulated. The study highlights the importance of surface chemistry in determining nanoplastic inflammatory potency. |
| 33. | Moorthy S et al. (2025) | Evidence, Mechanisms, and Clinical Implications of Microplastics and Nanoplastics As Emerging Cardiovascular Risk Factors: A Narrative Review | To narratively review the evidence, mechanisms, and clinical implications of microplastics and nanoplastics as emerging cardiovascular risk factors. | This study shows that MNPs promote cardiovascular disease through endothelial dysfunction, oxidative stress, inflammatory activation, platelet aggregation, and coagulation dysregulation, with human evidence from detection in atherosclerotic plaques and thrombi. Their small size enables penetration of biological barriers and accumulation in vascular tissues. The review argues for clinical awareness of MNP cardiovascular risk and calls for prospective human studies. |
| 34. | Ward J et al. (2023) | Nanotopography of Polystyrene/Poly(methyl methacrylate) for the Promotion of Patient Specific Von Willebrand Factor Entrapment and Platelet Adhesion in a Whole Blood Microfluidic Assay | To investigate nanotopography of polystyrene/poly(methyl methacrylate) surfaces for promoting Von Willebrand Factor entrapment and platelet adhesion in a whole blood microfluidic assay. | This study describes how the nanotopography of polymer surfaces significantly influenced von Willebrand Factor entrapment and subsequent platelet adhesion under flow conditions, with surface geometry modulating the thrombogenic response. Patient-specific differences in platelet adhesion were captured by the microfluidic model. The findings have implications for understanding how MNP surface properties influence thrombogenesis in vivo. |
| 35. | Sanfins E et al. (2014) | Size-dependent effects of nanoparticles on enzymes in the blood coagulation cascade | To investigate size-dependent effects of nanoparticles on enzymes in the blood coagulation cascade. | This study shows that nanoparticles of varying sizes differentially inhibited or activated coagulation cascade enzymes including thrombin and Factor Xa, with smaller particles showing greater disruption of enzymatic activity. The protein corona formed on nanoparticle surfaces was identified as a key mediator of these coagulation effects. The findings demonstrate that nanoparticles can fundamentally alter coagulation enzyme kinetics in a size-dependent manner. |

**Evidence of MNPs in Human Blood**

| **No.** | **Author** | **Title** | **Objective** | **Summary** |
| --- | --- | --- | --- | --- |
| 36. | Lee DW et al. (2024) | Microplastic particles in human blood and their association with coagulation markers | To assess microplastic particle concentrations in human blood and examine their association with coagulation markers. | Microplastic particles were detected in human blood samples with concentrations positively associated with elevated high sensitivity C-reactive protein and fibrinogen levels, both markers of systemic inflammation and procoagulant activity. Particle count correlated significantly with coagulation marker levels. The study provides direct human evidence linking circulating MNPs to a procoagulant inflammatory state. |
| 37. | Leslie HA et al. (2022) | Discovery and quantification of plastic particle pollution in human blood | Conducted an experimental study to detect and quantify plastic particles in human whole blood. The study also employed contamination controls and blank correction to ensure analytical reliability, reporting that plastic particles were present in nearly 80% of the samples, with polymer concentrations in the microgram-per-liter range. | This study illustrates that plastic particle pollution has entered the human bloodstream, providing direct evidence of internal human exposure to micro- and nanoplastic particles. By quantifying polymers in blood from a small cohort of healthy adults, the findings raise important questions about absorption, distribution, and potential health risks associated with plastic particles circulating in the human body. |

**Surface Modifications of MNPs as modulators of Virchow’s Triad**

| **No.** | **Author** | **Title** | **Surface Modification** | **Objective** | **Summary** |
| --- | --- | --- | --- | --- | --- |
| 38. | Rajendran D et al. (2023) | Journey of micronanoplastics with blood components | Protein corona | To review the journey of microplastics and nanoplastics through interactions with blood components, covering adsorption, cellular interactions, and biological consequences. | This study describes how MNPs interact extensively with blood components including proteins, red blood cells, platelets, and leukocytes, forming protein coronas that alter their surface properties and biological behaviour. These interactions promote hemolysis, platelet activation, coagulation cascade disruption, and immune cell activation. The review provides a comprehensive mechanistic account of MNP-blood interactions relevant to thrombotic and inflammatory risk. |
| 39. | Breznica P et al. (2020) | A review of the current understanding of nanoparticles protein corona composition | Protein corona | To review current understanding of nanoparticle protein corona composition and its implications for biological behaviour and toxicity. | This study points out that the protein corona forms rapidly upon nanoparticle exposure to biological fluids, with composition determined by particle physicochemical properties and the protein milieu. The hard corona determines long-term biological identity, while the soft corona is more dynamic. Protein corona composition fundamentally alters nanoparticle cellular interactions, biodistribution, and toxicity profiles. |
| 40. | Sun Y et al. (2024) | Protein Corona of Nanoparticles: Isolation and Analysis | Protein corona | To review methods for isolating and analysing the protein corona of nanoparticles, addressing methodological challenges and advances. | This study summarises multiple isolation techniques including centrifugation, size exclusion chromatography, and magnetic separation are used to characterize nanoparticle protein coronas, each with distinct advantages and limitations. Advances in mass spectrometry have enabled comprehensive proteomics-based corona characterization. Standardization of isolation and analysis methods is identified as critical for producing reproducible and comparable results across studies. |
| 41. | Nerland IL et al. (2014) | Report made for the Norwegian Environment Agency: Microplastics in marine environments; Occurrence, distribution and effects | Polyethylene, Polypropylene, Polyvinyl Chloride and Polystyrene | To review the occurrence, distribution, and effects of microplastics in marine environments. | Microplastics are ubiquitous in marine environments, detected from surface waters to deep sea sediments and in diverse marine organisms. They originate from fragmentation of larger plastic items and direct release of manufactured microplastics. The review documents early evidence of biological effects in marine species and identifies key research gaps in understanding ecological and food chain impacts. |
| 42. | Wang J et al. (2025) | Toxicological Impacts of Polypropylene Nanoparticles Similar in Size to Nanoplastics in Plastic-Bottle Injections on Human Umbilical Vein Endothelial Cells | Polypropylene | To investigate the toxicological impacts of polypropylene nanoparticles similar in size to nanoplastics on human umbilical vein endothelial cells. | This study discusses how polypropylene nanoparticles induced cytotoxicity, oxidative stress, and inflammatory responses in a concentration-dependent manner, impairing endothelial barrier function and promoting apoptosis. These effects were associated with disruption of tight junction proteins and activation of NF-κB signalling. The findings identify polypropylene nanoparticles as endothelial toxicants with implications for cardiovascular health. |
| 43. | Koner S et al. (2026) | Tracing the cellular consequences of polyethylene microplastics: senescence and apoptosis in A549 and Raw 264.7 macrophage cells | Polyethylene | To trace the cellular consequences of polyethylene microplastics in A549 and RAW 264.7 macrophage cells, focusing on senescence and apoptosis. | This study explains how polyethylene microplastics induced cellular senescence and apoptosis in both lung epithelial and macrophage cell lines, associated with DNA damage, mitochondrial dysfunction, and inflammatory cytokine release. Macrophages showed greater sensitivity to PE microplastics than epithelial cells. The findings suggest chronic PE microplastic exposure may accelerate cellular ageing and impair tissue homeostasis. |
| 44. | Yu H et al. (2026) | Microplastics release from infusion sets during intravenous infusion induces cardiovascular toxicity | Polyvinyl Chloride | To investigate microplastic release from infusion sets during intravenous infusion and assess the cardiovascular toxicity of these released particles. | This study mentions that microplastics were released from standard intravenous infusion sets during clinical use, with released particles (specifically polyvinyl chloride) inducing cardiovascular toxicity in experimental models including endothelial dysfunction, inflammatory activation, and cardiac stress markers elevation. The quantity of released particles increased with infusion duration and temperature. The findings highlight iatrogenic MNP exposure as a clinically significant and modifiable source of cardiovascular risk. |
| 45. | Nemmar A et al. (2003) | Size effect of intratracheally instilled particles on pulmonary inflammation and vascular thrombosis | Polystyrene | To investigate the size-dependent effects of intratracheally instilled particles on pulmonary inflammation and vascular thrombosis. | This study observes how intratracheal instillation of ultrafine particles induced greater pulmonary inflammation and vascular thrombosis compared to fine particles in a size-dependent manner. Ultrafine particles translocated from the lung to systemic circulation, promoting platelet aggregation and accelerating thrombosis in mesenteric vessels. The study provides early experimental evidence linking inhaled particle size to systemic thrombotic risk. |
| 46. | Sheng C et al. (2024) | Polystyrene nanoplastics enhance thrombosis through adsorption of plasma proteins | Polystyrene (Positive charge, negative charge and unmodified) | To investigate how polystyrene nanoplastics enhance thrombosis through adsorption of plasma proteins. | This study demonstrates that polystyrene nanoplastics adsorbed plasma proteins including fibrinogen and VWF, enhancing thrombosis through protein-mediated platelet activation and coagulation cascade amplification. The protein corona formed on nanoplastic surfaces directly promoted platelet aggregation and fibrin clot formation. The findings establish plasma protein adsorption as a key mechanism linking nanoplastic exposure to thrombogenesis. |
| 47. | Oslakovic C et al. (2012) | Polystyrene nanoparticles affecting blood coagulation | Polystyrene (Positive charge and negative charge) | To investigate the effects of polystyrene nanoparticles on blood coagulation, examining how particle exposure influences coagulation cascade activity. | This study demonstrates that polystyrene nanoparticles significantly affected blood coagulation by providing a procoagulant surface for assembly of coagulation complexes, accelerating thrombin generation and fibrin formation. Effects were dependent on particle size and surface area. The study identifies nanoparticle surface exposure as a driver of coagulation cascade activation relevant to thrombotic risk. |
| 48. | Kim EH et al. (2022) | Amine-modified nanoplastics promote the procoagulant activation of isolated human red blood cells and thrombus formation in rats | Polystyrene (Positive charge, negative charge and unmodified) | To investigate whether amine-modified nanoplastics promote procoagulant activation of human red blood cells and thrombus formation in rats. | This study shows that amine-modified nanoplastics induced phosphatidylserine externalization on red blood cell membranes, creating a procoagulant surface that activated the coagulation cascade and promoted thrombus formation in rat models. This effect was surface charge-dependent, with positively charged amine groups being key drivers of procoagulant activity. The findings demonstrate a novel mechanism by which surface-modified nanoplastics promote thrombosis through red blood cell modification. |
| 49. | Xu Y et al. (2024) | Photo-oxidation of Micro- and Nanoplastics: Physical, Chemical, and Biological Effects in Environments | Weathered MNPs | To review the physical, chemical, and biological effects of photo-oxidation on micro- and nanoplastics in environmental and biological contexts. | This study mentions that photo-oxidation alters the surface chemistry, size, and mechanical properties of MNPs, generating reactive oxygen species and increasing surface polarity and bioavailability. Weathered MNPs exhibit greater cellular toxicity and protein adsorption capacity compared to pristine particles. The review highlights environmental ageing as a critical factor in determining the biological hazard of MNPs. |
| 50. | Maitz MF et al. (2025) | Weathered microplastics in human blood: unraveling the effect of structural changes at the particle surface on coagulation and platelet activation | Weathered MNPs | To unravel the effects of structural changes at weathered microplastic surfaces on coagulation and platelet activation in human blood. | This study shows that weathered microplastics exhibited significantly enhanced procoagulant activity and platelet activation compared to pristine microplastics, attributable to increased surface roughness, oxidation, and altered protein corona composition. Weathering-induced surface changes promoted greater fibrinogen adsorption and coagulation factor binding. The findings demonstrate that environmental plastic ageing substantially amplifies thrombogenic risk. |
| 51. | Burke AR et al. (2011) | Determinants of the thrombogenic potential of multiwalled carbon nanotubes | Polyethylene Glycol | To determine the thrombogenic potential of multiwalled carbon nanotubes and identify key determinants of their procoagulant activity. | Multiwalled carbon nanotubes exhibited significant thrombogenic potential, promoting platelet activation and accelerating coagulation cascade activation in a surface chemistry- and length-dependent manner. Functional groups on nanotube surfaces were critical determinants of thrombogenicity. The study provides an early framework for understanding how nanoparticle surface properties drive thrombogenic risk applicable to MNPs. |

**Others**

| **No.** | **Author** | **Title** | **Objective** | **Summary** |
| --- | --- | --- | --- | --- |
| 52. | Rauert C et al. (2025) | Assessing the Efficacy of Pyrolysis–Gas Chromatography–Mass Spectrometry for Nanoplastic and Microplastic Analysis in Human Blood | To assess the efficacy of Pyrolysis–Gas Chromatography Mass Spectrometryfor detecting and quantifying nanoplastics and microplastics in human blood, identifying methodological limitations and realistic detection limits. | This study discusses how Pyrolysis–Gas Chromatography Mass Spectrometry is limited for detecting polyethylene and polyvinyl chloride in blood due to persistent lipid matrix interferences that generate false positives, even with advanced enzymatic digestion and double shot pyrolysis mode. Realistic detection limits were up to 20 times higher than nominal values, raising questions about biological plausibility of concentrations reported in prior studies. The method may be suitable for detecting polypropylene, polystyrene, and nylon in blood but requires rigorous quality control and complementary validation techniques. |
| 53. | Nihart AJ et al. (2025) | Bioaccumulation of microplastics in decedent human brains | To investigate the bioaccumulation of microplastics in decedent human brain tissue and compare concentrations across brain, liver, and kidney. | This study shows that microplastics were detected in human brain tissue at significantly higher concentrations than in liver and kidney, with polyethylene as the predominant polymer and concentrations appearing to increase in samples collected more recently. The findings suggest MNPs can cross the blood-brain barrier and accumulate preferentially in neural tissue. These results have been subject to methodological critique regarding potential lipid matrix interferences and quality control limitations. |
